# How Are Autism and Schizotypy Related? Evidence from a Non-Clinical Population

**DOI:** 10.1371/journal.pone.0063316

**Published:** 2013-05-15

**Authors:** Natalie L. Dinsdale, Peter L. Hurd, Akio Wakabayashi, Mick Elliot, Bernard J. Crespi

**Affiliations:** 1 Simon Fraser University, Burnaby, Canada; 2 University of Alberta, Edmonton, Canada; 3 Chiba University, Chiba, Japan; The University of Western Australia, Australia

## Abstract

Both autism spectrum conditions (ASCs) and schizophrenia spectrum conditions (SSCs) involve altered or impaired social and communicative functioning, but whether these shared features indicate overlapping or different etiological factors is unknown. We outline three hypotheses (overlapping, independent, and diametric) for the possible relationship between ASCs and SSCs, and compare their predictions for the expected relationships between autistic and schizotypal phenotypes using the Autism Spectrum Quotient and the Schizotypal Personality Questionnaire-Brief Revised from a large non-clinical sample of undergraduate students. Consistent with previous research, autistic features were positively associated with several schizotypal features, with the most overlap occurring between interpersonal schizotypy and autistic social and communication phenotypes. The first component of a principal components analysis (PCA) of subscale scores reflected these positive correlations, and suggested the presence of an axis (PC1) representing general social interest and aptitude. By contrast, the second principal component (PC2) exhibited a pattern of positive and negative loadings indicative of an axis from autism to positive schizotypy, such that positive schizotypal features loaded in the opposite direction to core autistic features. These overall PCA patterns were replicated in a second data set from a Japanese population. To evaluate the validity of our interpretation of the PCA results, we measured handedness and mental rotation ability, as these are established correlates of SSCs and ASCs, respectively. PC2 scores were significantly associated with hand preference, such that increasingly ‘schizotypal’ scores predicted reduced strength of handedness, which is consistent with previous research. PC1 scores were positively related to performance on the mental rotation task, suggesting trade-offs between social skills and visual-spatial ability. These results provide novel evidence for an autism-positive schizotypy axis, and highlight the importance of recognizing that psychological variation involving reduced social interest and functioning may have diverse causes.

## Introduction

The relationship between autism spectrum conditions (ASCs) and schizophrenia spectrum conditions (SSCs) has been subject to ongoing, unresolved investigation since Kanner [1] and Asperger [2] first defined and characterized autism [3]–[9]. Both spectra involve altered and impaired social and communicative functioning [10]–[12], which has suggested to some authors that autism and schizophrenia overlap in their etiologies [13]–[15]. One approach to addressing the relationship between ASCs and SSCs has been the analysis of psychometric, questionnaire data salient to both conditions, collected from clinical or non-clinical populations. Such data allow inferences to be drawn regarding patterns of cognitive-affective similarities and differences between ASCs and SSCs, which can provide insights into phenotypic overlap involving aspects of altered social functioning.

Impairments in social reciprocity, communication, and behavioural flexibility are central features of the autism spectrum [16]. Social and communication impairments are also characteristic of the schizophrenia spectrum, but these are not as central to the schizophrenia diagnosis as they are to autism. By contrast, symptoms and characteristics of schizophrenia can be understood as belonging to three dimensions: positive, disorganized, and negative. Positive symptoms involve hallucinations, paranoia, thought disorder, referential thinking, and delusions in schizophrenia, disorganized symptoms include bizarre speech, thoughts and behaviour, and negative symptoms capture social withdrawal, flattened affect, apathy and alogia [17], [18]. Of these phenotypes, negative symptoms appear most directly related to social and communication deficits, although disorganized behavior and positive symptoms may also involve social traits. It is also important to note that ‘negative’ refers to absences of normally-expressed phenotypes, whereas ‘positive’ refers to the presence of new phenotypes in schizotypy and schizophrenia; as such, both the disorganized and positive dimensions may involve ‘positive’ symptoms [18].

Since the delineation of autism from schizophrenia in the DSM-III [19], several studies have investigated overlap between ASCs and SSCs in various cognitive and behavioural features, most notably in the realm of social dysfunction. Konstantareas and Hewitt [20] contrasted the presenting characteristics of 14 men with high-functioning autism to 14 men with chronic paranoid schizophrenia using observational data, psychometric evaluations, medical records and comprehensive interviews. Their results revealed that no subjects with schizophrenia met the criteria for autism, almost half of the subjects with autism met criteria for disorganized schizophrenia, and the two diagnostic groups did not differ in their expression of negative schizophrenia symptoms including alogia, affective flattening, and attentional impairments, as assessed by the SANS (schedule for negative symptoms) [21].

In a non-clinical sample of college students, Hurst and colleagues [13] examined relationships between autistic features and schizotypal features using the Autism Quotient (AQ) [22] and the Schizotypy Personality Questionnaire (SPQ) [23] and found strong positive associations between total AQ score and total SPQ score. Correlations were especially strong between negative schizotypy and AQ social skills, and between disorganized schizotypy and AQ communication, though positive schizotypy was moderately and positively related to autistic-typal attention to detail and communication as well. The authors interpreted these trait associations as indicating that autism and schizophrenia may overlap on a single spectrum, rather than existing as two distinct spectra as currently implied by DSM categories.

Del Giudice and colleagues [24] reported a significant positive association between AQ and SPQ scores in a sample of young adults, especially between negative schizotypy and autistic-like interpersonal features. After statistically removing the overlap between the AQ and the SPQ, which was attributed to generality of wording and item similarity between questionnaires, the authors found that autistic and schizotpal traits predicted opposite patterns of self-reported behavior with regard to reproduction, which was taken as support for a diametric model.

Using the same self-report measures as Hurst et al. [13] and Del Giudice et al. [24] but with a clinical sample, Spek and Wouters [25] compared 21 adult males with autism to 21 adult males with schizophrenia and found that the presence or absence of negative schizophrenia symptoms could not reliably discriminate between the two conditions. For both diagnostic groups, negative schizophrenia symptoms were strongly and positively associated with autistic-like social and communication characteristics. The authors concluded that positive symptoms are the most useful in predicting presence of schizophrenia, whereas social skills are most predictive of autism.

In a related investigation, Wouters and Spek [26] assessed the ability of the AQ to differentiate between individuals with high-functioning autism, individuals with schizophrenia, and non-clinical individuals. They found that the AQ was 75% accurate in distinguishing subjects with autism from subjects with schizophrenia. The clinical groups did not show differences on attention to detail and imagination, but adults with autism reported a significantly more autistic-like social and communicative style than did the adults with schizophrenia. On a spectrum of autistic features, the authors suggested that healthy individuals and individuals with autism exist at opposing ends, and individuals with schizophrenia lie somewhere in between.

Using the AQ and the SPQ in a group of adolescents with and without ASCs, Barneveld and colleagues [27] found that subjects with ASCs were more likely to demonstrate schizotypal traits than subjects without an ASC diagnosis; the relationships were strongest for negative symptoms but extended to positive and disorganized symptoms as well. Given that AQ attention switching was related to all three forms of schizotypy, the authors suggested that attentional and executive functioning deficits might underlie some of the similarities in autistic and schizotypal phenotypes.

Russell-Smith et al. [28] sampled non-clinical adults using the Oxford-Liverpool Inventory of Feelings and Experiences (O-LIFE) [29] and the AQ, reporting positive correlations between total autism and total schizotypy scores. Positive schizotypy was also positively related to an empirically derived factor from the AQ that appeared to indicate autistic-typal recognition of pattern and detail. Consistent with Hurst et al. [13], the authors found the greatest overlap between the social deficits of autism and the interpersonal symptoms of schizotypy. They interpreted their results as consistent with models positing common factors for the social deficits and alterations shared between ASCs and SSCs.

Wakabayashi et al. [30] studied the relationships between autism, schizotypy, and obsessive-compulsive disorder (OCD) and, like Hurst et al. [13] and Russell-Smith et al. [28], found modest, positive correlations between the SPQ and the AQ, with the most overlap between autism and schizotypy in the area of social-affective difficulties. In a multiple regression, however, positive schizotypy did not predict autistic features. Overall, the authors concluded that autism and schizophrenia share more differences than they do similarities.

These previous studies provide empirical support for phenotypic overlap between ASCs and SSCs at the psychometric and behavioural levels in both clinical and non-clinical populations. Some studies have reported overlap of autistic features with manifestations of positive schizotypy [13], [27], [28], but the strongest overlap between the two spectra consistently appears between the negative symptoms of SSCs and the social-communicative deficits that are central to ASCs [13], [20], [25], [27], [28], [30]. Generalized deficits such as abnormal social functioning comprise a broad range of phenotypes and likely reflect a multitude of complex causal factors, such that phenotypic similarities inferred from psychometric and behavioural data are not sufficient to infer shared etiologies [31].

In this study we used two of the primary measures of ASCs and SSCs, the Autism Spectrum Quotient and a revised version of the Schizotypy Personality Questionnaire-Brief, to evaluate hypotheses for the relationship between these sets of conditions. Given that various perceptual, cognitive, and affective alterations underlie the behavioural abnormalities expressed in ASCs and SSCs, we also measured two phenotypic correlates of autism and schizophrenia, mental rotation ability and strength of handedness, in order to provide validation of our interpretation of the questionnaire data analyses. First, previous research has demonstrated enhanced visual-spatial abilities, such as superior performance on mental rotation tasks, in individuals with autism and in individuals with autistic features [32]–[36]. More broadly, autistic islets of visual-spatial ability may stem from enhancements in primary perceptual functions in ASCs; importantly, these perceptual alterations may underlie the social and communication features viewed as central to the spectrum [37]. Within the schizophrenia spectrum, mental rotation performance appears reduced and strongly affected by sex: on a classic mental rotation task [38], Jiménez et al. [39] found that males with schizophrenia performed worse than females with schizophrenia and worse than the control groups, whereas females with and without schizophrenia did not differ significantly in their performance. In women only, Thakker and Park [40] found that low negative schizotypy predicted slower rotation of 2-dimensional letters. In a large sample of adults with schizophrenia, Zhai et al. [41] found that two SNPs associated with increased schizophrenia risk predicted reduced mental rotation performance in the clinical group. Taken together, enhanced performance on mental rotation tasks is associated with the autism spectrum whereas relatively reduced performance on similar tasks may, at least somewhat, characterize aspects of the schizophrenia spectrum.

Second, atypical patterns of cerebral asymmetry appear to play a role in schizophrenia etiology, and multiple studies have reported decreased cerebral lateralization, as indexed by a handedness preference leaning toward mixed and non-right handedness, in both schizotypy and schizophrenia [42]–[48]. Mixed handedness appears to be particularly predictive of positive schizotypal features such as magical thinking and delusional beliefs [49]–[52]. In previous studies that used the Waterloo Handedness Questionnaire (WHQ) [53] to examine hand preference in schizotypy and schizophrenia, Upadhyay et al. [54] found an increased prevalence of mixed handedness in individuals with schizophrenia, and Bryson et al. [55] reported that mixed handedness predicted magical ideation. In autism, some studies have indicated ambiguous (no clear hand preference) handedness [56]; dissociation between hand skill and hand preference [57]; or differences in handedness patterns as a function of specific autistic subtypes [58], but no clear patterns have emerged. Overall, handedness is a well-validated and easily assessed correlate of SSCs, especially for the positive dimensions of schizophrenia.

Most studies comparing ASCs with SSCs using psychometric data have focussed on single, specific predictions, rather than testing a set of alternative hypotheses. Here, we use autism and schizotypy questionnaires in addition to data from mental rotation performance and handedness preference to evaluate three hypotheses for the relationship between the autism spectrum and the schizophrenia spectrum. This approach allows us to validate our interpretations of the relationship between autistic and schizotypal features in in order to build explicit models of how the two disorder spectra may be related to one another at both etiological and phenotypic levels.

Firstly, observations of distinct developmental trajectories for autism and schizophrenia [3] and the absence of evidence for an increased prevalence of schizophrenia in autistic populations [59] have shaped the view that the etiologies underlying the two disorders are independent [4]. This perspective is implied by DSM IV-TR categories, which treat autism and schizophrenia as mutually exclusive diagnoses [60]. Under this ‘independence’ model, we would expect that autistic features, as measured by scores on Baron-Cohen and colleagues’ AQ [22], should not be strongly positively associated with scores on aspects of schizotypy. Additionally, we would predict that mental rotation ability predicts autistic features whereas handedness predicts schizotypal features. From this perspective, any similarities between the two spectra may be attributable to different causal factors.

Secondly, shared phenotypes between autism and schizotypy could reflect overlapping etiologies; as also suggested by recent genetic evidence interpreted as indicating shared risk factors between autism and schizophrenia [61], [62]. Both spectra involve reduced performance across a range of social-cognitive abilities, including theory of mind and emotion recognition, as well as corresponding impairments in overall social functioning [63]–[66]. By this ‘overlap’ model, if shared factors underlie the overlapping social alterations and impairments between autism and schizotypy, then autistic features and schizotypal features should show positive associations for those phenotypes specifically related to social interests and social functioning. Furthermore, we may predict that neither mental rotation performance nor hand-preference associate uniquely with the questionnaire data.

Thirdly, Crespi and Badcock [67] forwarded the ‘diametric disorders’ hypothesis, which places autism and schizotypy on opposite ends of an axis of social cognition. By this ‘diametric’ model, the phenotypes of autism spectrum centrally involve under-developed social cognition, whereas positive schizophrenia spectrum phenotypes involve manifestations of over-developed social cognition, such as ‘hyper-developed’ theory of mind in paranoia or an exaggerated sense of self and agency in megalomania [67]. Under the diametric model, social deficits and abnormalities as assayed by psychometric data or psychological tests could manifest similarly (for example, as social withdrawal, disinterest, or skill reductions), but such similarities may reflect highly diverse or diametrically different causes between ASCs and SSCs. The diametric model predicts that when variation due to general social deficits, alterations and abnormalities is removed, an autism-schizotypy phenotypic axis will emerge where autistic features negatively predict positive schizotypal features. Under the diametric model, we would also expect that the autism-positive schizotypy axis associate with both handedness and mental rotation, such that increasing schizotypy predicts reduced lateralization and increasing autism predicts increased mental rotation performance.

We evaluated predictions from the independent, overlap and diametric models through (1) examining the bivariate correlations between subscales of the AQ and a revised version of Raine and Benishay’s [68] brief Schizotypal Personality Questionnaire (SPQ-BR) [69], (2) performing a principal components analysis (PCA) on subscale scores from the AQ and SPQ-BR, and (3) relating the questionnaire data to strength of handedness and performance on a mental rotation task. Together, these analyses provide an unbiased assessment of the phenotypic structures of autism and schizotypy as measured using two of the primary questionnaires deployed in ongoing research, and they allow us to link neuropsychological correlates of either spectra with patterns of overlap between spectra. Additionally, this approach allows us to determine in particular if an autistic-positive schizotypy axis emerges, as predicted under the diametric model.

## Methods

### Sample and Ethics Statement

Data was collected from 605 undergraduate students (380 females and 225 males) at both University of Alberta and Simon Fraser University. As part of a larger collaborative project approved by Human Research Ethics at University of Alberta and by the Simon Fraser University Research Ethics Board, data was collected in single sessions held over the course of two semesters at each institution. All students gave written consent before participating in the study.

### Measures

We used the Autism Spectrum Quotient (AQ) [22] to measure the extent to which participants endorsed traits consistent with the autism spectrum. The AQ is a brief self-report questionnaire that provides a quantitative measure of autism-related psychological traits in adults of normal intelligence [70]–[72]. A total of 50 questions assess autistic traits across five areas (10 questions per domain) which include: 1) *social skills*; 2) *communication*; 3) *attention to detail*; 4) *attention switching*; and 5) *imagination*. For the *social skills* and *communication* domains, either poor social skills and communication or the preference to be in non-social situations increases the participant’s score. Attention shifting tends to be less flexible in autism [73], [74], so if participants describe their attention as focused or inflexible, they are scored by the questionnaire as ‘more autistic’. Similarly, individuals with autism tend to demonstrate heightened attention to detail and interest in activities requiring such resources [75]–[77] and therefore, responses endorsing these attributes increase the AQ score. Responses are in a 4-point Likert-scale format ranging from ‘definitely agree’ to ‘definitely disagree’, and whenever participants mildly or strongly endorse a trait in the autistic direction, they score one point for that question (taking into account reverse-scored items) for a possible scoring range of 0–50.

Schizotypy was assessed using the Schizotypal Personality Questionnaire-Brief Revised (SPQ-BR) [69]. This instrument is a revised version of the SPQ-Brief [68] and includes 32 items in a 5-point Likert-scale format with response choices ranging from ‘strongly disagree’ to ‘strongly agree’. Scores can range from 0 to 160 with increasing scores reflecting higher levels of schizotypy. Factor analysis on the SPQ-BR has supported seven subscales that cluster satisfactorily into three or four super-ordinate factors. These three higher-order factors include *cognitive-perceptual* schizotypy, *interpersonal* schizotypy, and *disorganized* schizotypy; these factors map onto the positive, negative, and disorganized dimensions of schizophrenia as described above [17], [18], [78]. Under the factor of cognitive-perceptual (positive) schizotypy, there are three subscales: *magical thinking; unusual perceptions*; and *ideas of reference*. For interpersonal (negative) schizotypy, the subscales include *constricted affect* and *social anxiety*. The disorganized factor includes *eccentric behaviour* and *odd speech*. This measure was chosen because it is brief, sensitive, and covers a broad range of schizotypal features.

We also gave subjects the Mental Rotation Test (MRT) [79] and the Waterloo Handedness Questionnaire (WHQ) [53] as these measures tap into cognitive and perceptual factors that likely underlie autistic and schizotypal symptomatology. The MRT is a timed 20-item test that requires participants to mentally rotate three-dimensional objects to choose one figure, out of four possible options, that correctly matches the target object. The WHQ is a 32-item questionnaire asking participants to identify which hand they use for a variety of tasks. Response choices range from strongly left to either hand to strongly right with overall scores ranging from −32 to +32. We used absolute values of WHQ scores to measure the strength of handedness irrespective of direction, given that strength is a relatively strong predictor of schizotypy [46].

Data from Wakabayashi et al. [30] were also included for use as a replication data-set; in this study, the authors gave the AQ and Raine’s [23] original 74-item SPQ to a large sample (n = 662) of non-clinical adults. To provide a fully unbiased assessment of the phenotypic structure of the autism and schizotypy data, we performed a principal component analysis (PCA) on the AQ and the SPQ-BR, for the data collected here in addition to the data from Wakabayashi et al. [30]. Given that the AQ is scored on a different and smaller scale than the SPQ-BR, we *a priori* chose to use the correlation matrix rather than the covariance matrix in the PCA; therefore, the factor loadings are expected to be lower and more similar to one another compared to output using covariance matrix and additional rotations. All analyses were performed in R.

## Results

Data were analyzed from 605 participants (380 females and 225 males) with a mean age of 19.4 years. The majority of study subjects identified their ethnicity as Western European descent (87%) and ethnicity did not show significant effects in any of the following analyses. A summary of descriptive statistics and sex differences for the four measures (AQ, SPQ-BR, MRT, WHQ) is presented in Table 1.

**Table 1:** Descriptive statistics and sex differences in AQ, SPQ-BR, mental rotation and handedness.

Measure		Males (*n = 225*)		Females (*n = 380*)		Welch’s t-test		
		mean	S.D.	mean	S.D.	*t*	*df*	*p*
**AQ**	Social skills	2.57	1.68	2.44	1.70	0.92	474.5	0.36
	Attention switching	4.94	2.10	4.63	1.96	1.79	444.3	0.07
	Attention to detail	5.40	2.29	5.38	2.11	0.10	439.6	0.92
	Communication	2.52	1.93	2.11	1.80	2.59	444.9	<0.05
	Imagination	2.51	1.70	2.11	1.58	2.88	442.0	<0.01
	***Total AQ***	17.93	5.84	16.66	5.15	2.70	424.4	<0.01
**SPQ-BR**	Social anxiety	11.17	3.64	10.64	3.76	1.72	482.9	0.09
	Constricted affect	15.38	4.87	13.98	4.83	3.44	467.0	<0.001
	**Interpersonal**	26.56	7.22	24.62	7.27	3.18	472.7	<0.01
	Eccentric behaviour	12.45	3.97	11.11	3.87	4.05	460.8	<0.001
	Odd speech	9.85	2.28	10.22	2.48	−1.85	502.7	0.06
	**Disorganized**	22.30	5.27	21.33	5.35	2.19	476.5	<0.05
	Magical thinking	7.58	3.36	8.23	3.57	−2.25	494.1	<0.05
	Unusual perceptions	10.33	3.21	9.52	2.94	3.12	438.2	<0.01
	Ideas of references	17.22	4.31	16.60	4.36	1.71	474.8	0.09
	**Cognitive-perceptual**	35.14	7.85	34.35	8.24	1.17	488.7	0.24
	***Total SPQ-BR***	83.99	15.12	80.29	15.88	2.85	488.9	<0.01
**MRT**		12.62	5.00	9.11	4.49	8.66	431.1	<0.001
**WHQ** 1		34.08	21.72	36.71	24.43	−1.25	429.9	0.21

1 ranges from −64 to +64. Negative scores indicate left-handedness, positive scores indicate right-handedness, higher scores indicate stronger handedness, and scores closer to 0 indicate mixed handedness.

Total AQ score was positively and significantly correlated with total SPQ-BR score in males, females, and in both sexes when analyzed together (Table 2). This positive relationship between the AQ the SPQ-BR was consistent for most of the subscales. Of the AQ scales that deviated from this general pattern, *attention to detail* was negatively correlated with SPQ-BR *odd speech* in females only, and *imagination* was negatively correlated with SPQ-BR *magical thinking* in males and in both sexes when analyzed together.

**Table 2:** Correlation coefficients between AQ and SPQ-BR in males only, females only, and males and females combined.

	AQ																	
	Social skills			Attention switching			Attention to detail			Communication			Imagination			*Total AQ*		
SPQ-BR	m	f	m+f	m	f	m+f	m	f	m+f	m	f	m+f	m	f	m+f	m	f	m+f
Social anxiety	0.55**	0.47**	0.50**	0.43**	0.40**	0.41**	0.01	0	0	0.37**	0.44**	0.42**	0.24**	0.11†	0.17**	0.51**	0.5**	0.5**
Constricted affect	0.41**	0.41**	0.41**	0.14†	0.13†	0.14**	0.06	0.02	0.03	0.33**	0.41**	0.39**	0.15†	0.12†	0.15**	0.34**	0.37**	0.37**
**Interpersonal**	0.55**	0.52**	0.53**	0.31**	0.29**	0.3**	0.04	0.01	0.02	0.4**	0.5**	0.47**	0.22**	0.14*	0.18**	0.48**	0.5**	0.5**
Eccentric behaviour	0.24**	0.26**	0.25**	0.21*	0.05	0.13*	0.27**	0.01	0.12*	0.37**	0.3**	0.34**	0.04	0	0.04	0.38**	0.22**	0.3**
Odd speech	0.06	0.15*	0.12*	0.25**	0.14*	0.17**	0.13†	−0.14*	−0.04	0.21*	0.27**	0.24**	−0.02	−0.02	−0.03	0.22**	0.14*	0.16**
**Disorganized**	0.2*	0.26**	0.24**	0.27**	0.1†	0.17**	0.26**	−0.05	0.07	0.36**	0.34**	0.35**	0.02	0	0.01	0.38**	0.22**	0.29**
Magical thinking	−0.12	0	−0.04	0	0	0	0.12	0.09	0.1†	−0.05	0.06	0.01	−0.16†	−0.06	−0.1*	−0.05	0.04	0
Unusual perceptions	0.03	0.09	0.07	0.12	0.14*	0.14**	0.14†	0.09	0.11*	0.07	0.22**	0.17**	−0.05	−0.06	−0.04	0.11	0.17**	0.16**
Ideas of reference	0.2*	0.13†	0.16**	0.3**	0.24**	0.26**	0.11	0.08	0.09†	0.26**	0.25**	0.26**	0.01	−0.07	0.06	0.3**	0.28**	0.29**
**Cognitive-perceptual**	0.08	0.1	0.09†	0.21*	0.17**	0.19**	0.17†	0.11†	0.13**	0.14†	0.23**	0.2**	−0.08	0	−0.03	0.19*	0.22**	0.21**
***Total SPQ-BR***	0.36**	0.36**	0.36**	0.34**	0.25**	0.29**	0.19*	0.04	0.1†	0.38**	0.45**	0.43**	0.07	0.05	0.07	0.45**	0.41**	0.43**

† denotes significance at <0.05;

* denotes significance at <0.01;

** denotes significance at <0.001.

A principal components analysis (PCA) was performed using the R *prcomp* function on the correlation matrix of scores for the five subscales of the AQ (s*ocial skills, attention switching, attention to detail, communication,* and *imagination*) and the seven subscales of the SPQ-BR (*social anxiety, constricted affect, eccentric behaviour, odd speech, magical thinking, unusual perceptions,* and *ideas of reference*). Given that the AQ is scored on a smaller scale than the SPQ-BR, we chose to standardize the data by using the correlation matrix rather than the covariance matrix. We included the seven subscales of the SPQ-BR in the PCA rather than their higher-order factors (*positive/cognitive-perceptual, negative/interpersonal, disorganized*) because collapsing the subscales together would obscure the contributions of specific subscales (e.g. *magical thinking)* that demonstrated unique associations with the AQ in the correlation tests; this decision allowed for a finer-grained analysis in addition to providing output that builds upon the factor analysis on AQ and O-LIFE data in Russell-Smith and colleagues [28].

The patterns and strengths of loadings for PC1 and PC2 were directly interpretable, so we did not perform any rotations on the components (note that performance of PCA using correlation matrices results in smaller-magnitude loadings than does analysis using covariance matrices). Table 3 shows the loadings of each subscale on components 1 and 2 for both sexes combined; we performed sex-specific PCAs as well, but the differences between the females and males were minor and therefore the presented data and interpretations of the data are from the combined sex analysis only. Principal component 1 (PC1) demonstrated positive loadings with both AQ and the SPQ-BR domains, and accounted for 29.2% of the variance, whereas principal component 2 (PC2) included positive and negative loadings from both questionnaires, and accounted for 14.7% of the variance. Given that each of the remaining components explained a small quantity of the total variation (<8%) and that we did not make any predictions concerning their interpretation, only PC1 and PC2 are included in Tables 3 and 4 and considered here. Males scored significantly higher than females on PC1 (x_males_ = 0.33, x_females_ = −0.14; t = 3.15, df = 472.92, p = 0.002). Females scored slightly higher than males on PC2 (x_females_ = 0.076, x_males_ = −0.079) but this difference did not approach statistical significance (t = −1.37, df = 454.52, p = 0.16).

**Table 3:** Principal components 1 and 2 loadings across the AQ and SPQ-BR subscales for females and males combined.

Measure		PC1 (29.2%)	PC2 (14.7%)
**AQ**	Social skills	0.334	−0.364
	Attention switching	0.269	−0.205
	Attention to detail	0.040	0.166
	Communication	0.364	−0.242
	Imagination	0.132	−0.349
**SPQ-BR**	Social anxiety	0.390	−0.177
	Constricted affect	0.346	−0.072
	Eccentric behaviour	0.338	0.188
	Odd speech	0.273	0.273
	Magical thinking	0.129	0.488
	Unusual perceptions	0.278	0.412
	Ideas of reference	0.331	0.251

**Table 4:** Wakabayashi et al. (2012) PCA results from SPQ* and AQ in males and females.

Measure		PC1 (28%)	PC2 (16.8%)
**AQ**	Social skills	0.364	−0.275
	Attention switching	0.326	−0.045
	Attention to detail	0.082	0.286
	Communication	0.371	−0.130
	Imagination	0.268	−0.222
**SPQ**	Social anxiety	0.330	−0.024
	Constricted affect	0.334	−0.127
	No close friends^1^	0.345	−0.162
	Suspiciousness^2^	0.262	0.295
	Eccentric behaviour	0.205	0.246
	Odd speech	0.195	0.230
	Magical thinking	−0.031	0.394
	Unusual perceptions	0.146	0.409
	Ideas of reference	0.186	0.456

**Notes**

* SPQ (Raine 1991) has 2 more subscales than the SPQ-BR, for a total of 9 subscales.

1 Part of *interpersonal* subscale.

2 Part of *interpersonal* and *cognitive-perceptual* subscales.

PC1 demonstrated relatively strong positive loadings for two of the AQ subscales (*social skills* and *communication*) and for four of the SPQ-BR subscales (*social anxiety, constricted affect, eccentric behaviour,* and *ideas of reference*). PC2 included relatively strong negative loadings from two of the AQ subscales (*social skills* and *imagination*) and especially strong positive loadings from two of the seven SPQ-BR subscales (*magical thinking* and *unusual perceptions*). Most of the AQ subscales and SPQ-BR subscales were positively related and a few were negatively related. The positive correlations between several of the AQ and SPQ-BR subscales are reflected in the PCA output; PC1 demonstrated positive loadings from several domains, with the strongest loadings from subscales related to social and communicative functioning (AQ: c*ommunication, social skills*; SPQ-BR: *social anxiety, constricted affect, eccentric behaviour, ideas of* reference). These results are inconsistent with independence between autistic and schizotypal features as assayed by these metrics.

**Figure 1:**
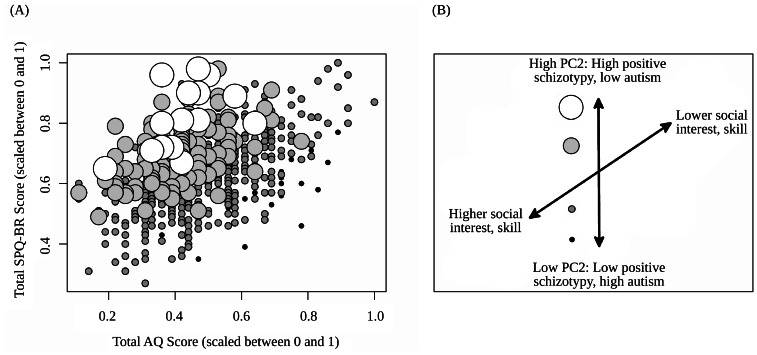
PC2 as an axis from autism to positive schizotypy. *****a)***** Plot of AQ scores versus SPQ-BR scores with point size indicating relative PC2 score (point size increases as PC2 score increases) and a ***b)*** schematic representation of the relationships between AQ scores, SPQ-BR scores, and PC2 variation.

For all loadings stronger than 0.20 on PC2, subscales from the AQ loaded negatively and subscales from the SPQ-BR loaded positively. Given that the SPQ-BR subscales that loaded oppositely to AQ subscales were from the positive/cognitive-perceptual and disorganized schizotypy factors, the pattern of PC2 loadings can be interpreted as an axis where aspects of positive dimensions of schizotypy are diametric to autistic features. Consistent with this interpretation is the finding that PC2 was negatively associated with total AQ score (r = −0.43, p<0.0001) and positively associated with total SPQ-BR score (r = 0.32, p<0.0001). Figures 1a and 1b depict and describe the relationships between the AQ, SPQ-BR, and PC2.

PCA results for the AQ and SPQ data from Wakabayashi et al. [30] (Table 4) were similar to the PCA results shown in Table 3. The first component explained 28% of the variance and indicated relatively strong positive loadings for AQ *social skills, attention switching,* and *communication* and for SPQ *social anxiety, constricted affect, no close friends*. The second component accounted for 16.8% of the variance and demonstrated negative loadings for all AQ subscales (except for *attention to detail*). As in Table 3, especially strong negative loadings from the AQ were evident on PC2 for *social skills* and *imagination,* contrasting with relatively strong positive loadings for subscales belonging to positive/cognitive-perceptual and disorganized schizotypy (highest for *ideas of reference, unusual perceptions,* and *magical thinking*).

### Autism, Schizotypy, and Mental Rotation

The questionnaire results were then tested for association with scores on the mental rotation task (MRT), given that previous research has provided evidence for enhanced MRT performance in individuals with autism and in individuals with autistic features [35]. We found that total AQ score, as well as *social skills*, positively predicted MRT score in both sexes, though the positive correlation between MRT and total AQ score was driven by males (Table 5). AQ a*ttention switching* and MRT score were also positively associated, in males only. We found positive correlations of MRT performance with *negative/interpersonal* and *disorganized* schizotypy and negative associations of MRT score with *ideas of reference* (in females) and m*agical thinking* (in males) (Table 5). PC1 demonstrated a significant positive correlation with MRT score in both sexes, though males drove this effect; PC2 was not related to MRT performance (Table 5).

**Table 5:** Correlation coefficients of AQ, SPQ-BR, PC1 and PC2 with mental rotation and absolute handedness^1^.

		Mental Rotation Score					Absolute WHQ Score^1^			
Measure		males		females	m+f		males	females		m+f
**AQ**	**Social skills**	0.107		0.091	0.101†		0.010	−0.045		−0.028
	**Attention switching**	0.146†		−0.062	0.044		0.070	0.047		0.052
	**Attention to detail**	0.049		0.031	0.036		−0.038	−0.033		−0.033
	**Communication**	0.047		−0.014	0.046		−0.054	0.051		0.010
	**Imagination**	−0.020		0.002	0.033		−0.060	0.044		−0.003
	**Total AQ**	0.112		0.016	0.088†		−0.020	0.018		−0.005
**SPQ-BR**	**Social anxiety**	0.112		0.014	0.070		0.020	−0.060		−0.002
	**Constricted affect**	−0.010		0.032	0.062		−0.137*	−0.045		−0.082
	***Interpersonal***	0.052		0.029	0.077†		−0.087	−0.036		−0.059*
	**Eccentric behaviour**	0.147†		0.131*	0.180**		−0.083	−0.057		−0.085*
	**Odd speech**	0.041		0.037	0.014		−0.053	−0.042		−0.037
	***Disorganized***	0.127†		0.111†	0.138**		−0.097	−0.062		−0.081*
	**Magical thinking**	−0.181*		−0.052	−0.124*		−0.082	−0.105*		−0.084*
	**Unusual perceptions**	0.073		−0.016	0.062		−0.094	−0.016		−0.052
	**Ideas of references**	0.033		0.114†	−0.031		−0.068	−0.031		−0.041
	***Cognitive-perceptual***	−0.028		−0.087	−0.045		−0.102†	−0.062		−0.074†
	**Total SPQ-BR**	0.053		0.005	0.057		−0.120†	−0.062		−0.086*
**PC1**		0.13†	0.03			0.11*	−0.04		0.01	−0.01
**PC2**		0.05	0.01			0.03	−0.10†		−0.06	−0.08†

† denotes significance at <0.05;

* denotes significance at <0.01;

** denotes significance at <0.001.

1 All handedness correlation coefficients are Kendall’s tau.

### Autism, Schizotypy, and Strength of Handedness

Because one of the four study cohorts did not complete the Waterloo Handedness Questionnaire (WHQ), the sample size for all analyses using this data is n = 504 (316 females and 188 males). The distribution of WHQ scores was strongly skewed, so we used a Kendall’s rank correlation test for all following analyses. Consistent with previous research, total SPQ-BR score as well as all three subtypes of schizotypy (*interpersonal/negative*, *disorganized*, and *cognitive*-*perceptual/positive*) were significantly and negatively associated with strength of handedness (Table 5). Handedness strength did not demonstrate any correlations with the AQ. PC2 was negatively associated with strength of handedness (Table 5).

## Discussion

This study examined autistic and schizotypal features in relation to each other, and to mental rotation performance and handedness strength, in a large non-clinical sample to test specific predictions from the independent, overlap, and diametric models for the relationship between ASCs and SSCs. Drawing from correlation tests and PCA results, we found support consistent with both the overlapping and diametric models, and no evidence for the model of independence. Overall, our results are consistent with previous research indicating a large degree of phenotypic overlap between autism and schizotypy, and novel in revealing support for an autism-positive schizotypy phenotypic axis, but only after the effects of general social alterations and abnormalities are removed.

In accordance with results from several previous studies [13], [28] we found evidence indicating considerable overlap between autistic and schizotypal features that are related to interest and aptitude in social and communicative functioning. Autistic-like communication and social skills were most strongly and positively associated with *interpersonal* (negative) schizotypy, followed by *disorganized* schizotypy. These positive correlations were evident in PC1: this component included relatively-strong positive loadings from subscales of both the AQ and the SPQ-BR, and the strongest loadings were found for *social skills, communication, social anxiety, constricted affect, eccentric behaviour,* and *ideas of reference*. Consistent with both the overlapping and diametric models, we interpreted PC1 as a factor indicating general social-communicative disinterest, impairments and abnormalities. The subscale *attention to detail* demonstrated a different pattern of loadings in comparison to the other AQ subscales (very low and positive on PC1, moderately-low and positive on PC2). According to Baron-Cohen et al. [22], *attention to detail* measures a person’s tendency and preference to focus on and recall local rather than global details (e.g. Item 5: “I notice small sounds when others do not”). Detail-oriented attention may represent a separate dimension of autism, relatively independent of its other features, as also reported by Hoekstra et al. [72] and Hurst et al. [80].

The overlap between autistic and schizotypal features evidenced in PC1 is consistent with the high degree of conceptual, diagnostic, and phenotypic overlap observed between the broader social phenotypes of autism and schizophrenia, especially between Asperger’s syndrome and schizoid personality in childhood and/or schizotypal personality disorder (SPD) [7], [6], [81]. Both Asperger’s syndrome and SPD are characterized by reduced social abilities, odd or eccentric social behaviour, mental rigidity, and unusual communication, which has led to questions whether these two higher-functioning forms of disorder represent discrete or overlapping conditions [81]. Given that altered social functioning may involve a diversity of complex causes, evidence for shared behavioural phenotypes involving weaknesses or general abnormalities has limited ability to explain how or why such similarities exist [31]. Future research investigating patterns of similarities and differences in the specific nature of social abnormalities between ASCs and SSCs will be useful in further understanding the relationship between these sets of conditions. For example, Konstantareas & Hewitt [20] observed thought disorder in both high-functioning autism and paranoid schizophrenia, but they described the disordered thoughts of individuals with autism as relatively ‘immature’ in comparison to the complex and bizarre thoughts of the subjects with schizophrenia. Indeed, the overlap between autistic social-communicative deficits and schizotypal interpersonal features may reflect the insensitivity of psychological questionnaires to pick up on the differences in the nature of the social alterations that may be characteristic of each spectrum, an important consideration also discussed by Del Giudice and colleagues [24]. Our findings and these considerations are not inconsistent, however, with the hypothesis that the presence of social deficits contributes to risk or expression of both autism spectrum and schizophrenia spectrum disorders.

The diametric model and the overlapping model do not diverge in their predictions with respect to PC1, but the models differ in their explanation for the causes underlying the similarity: the overlapping model suggests that shared causal factors underlie the social traits common to both the autism spectrum and schizophrenia spectrum, whereas the diametric model suggests that opposite causal factors lead, to some degree, to general social phenotypes that present similarly enough to show strong correlations. Under the diametric model, the overlap evidenced by the bivariate correlations and PC1 need not reflect shared causal factors, but rather may indicate that autism and schizotypy involve social deficits and abnormalities due to different causes. By this hypothesis, social deficits and abnormalities in autism may represent central, underlying causes of the autism-related traits, whereas social deficits and abnormalities in schizotypy (and, by extension, in schizophrenia) may predominantly represent effects of the neuropsychological alterations that characterize the schizophrenia spectrum, including paranoia, alterations to regulation of affect, social ambivalence, social anhedonia, delusions with social content, and ideas of reference, as well as impaired social cognition due to general cognitive deficits and disorganized thought processes. Some of these psychological alterations, especially paranoia, delusions, and ideas of reference, involve aspects of hyper-mentalizing [82], [83] and illustrate how peculiar social behaviour and social withdrawal in schizophrenia may result from causes that are diametric to those underlying autistic social deficits, which are present from early childhood and are associated with other phenotypes (such as resistance to change, detail-focussed attention, and visual-spatial abilities enhanced relative to verbal skills), as described by Kanner [1] and summarized in Crespi [8].

Consistent with a prediction specific to the diametric model, we found evidence consistent with a phenotypic axis of autism-positive schizotypy (PC2), after removing apparent general social-deficit and abnormality variation measured by both questionnaires and quantified by PC1. Based on the pattern of PC2 loadings, this autism-schizotypy axis appears to reflect a diametric pattern between autistic features and positive aspects of schizotypy (including both ‘positive’ and ‘disorganized’ dimensions), most strongly manifesting in magical thinking and unusual perceptions. The presence of such as axis was consistent with the finding that PC2 was negatively associated with absolute strength of handedness across individuals, such that increased PC2 scores (in the ‘schizotypal’ direction) predicted reduced lateralization. This effect was small, though it is similar in magnitude to many other findings in handedness research [84]. Additional test for associations of PC2 scores with variables that are well-established correlates of schizophrenia, schizotypy, and autism will be useful in further evaluating the hypothesis that this score quantifies an axis from autism spectrum, to normality, to positive schizotypy. More generally, confirmatory factor analysis could also be applied to test specific alternative models, as derived from our results.

We compared questionnaire scores to performance on a mental rotation task, and did not find a relationship between MRT skill and PC2 scores, as we would expect under the diametric model. However, PC1 was significantly and positively related to MRT score across the entire sample, though analyzing the sexes separately indicated that males drove this effect. Autistic and schizotypal subscales that also positively predicted MRT included: social skills; *attention switching* (males only); total AQ score (significant in entire sample, though driven by males); *interpersonal* (*negative*) schizotypy; and *disorganized* schizotypy. Taken together, these results suggest that enhanced mental rotation skills are associated with reduced social-communicative functioning; this is an interesting finding that may be explicable in the context of trade-offs between different types of cognitive systems [85] or trade-offs between verbal compared to visual-spatial skills more generally [86], [87]. Links between altered social skills and enhanced visual-spatial ability warrant further investigation, especially with respect to sex-specific effects.

Our study has several limitations. First, undergraduate samples may not be characteristic of the population at large, especially with regard to children and mature adults. Second, some of the correlation coefficients are low in magnitude, although statistically significant. Third, whether or not the results that we obtained through comparing AQ scores to SPQ-BR scores are generalizable to other widely-used psychometric instruments for assessing autism and schizotypy, such as the Social Responsiveness Scale (SRS) [88] and the O-LIFE [29], remains unknown. Fourth, self-report data alone is constrained in its ability to provide a detailed picture of the relationship between autism and schizotypy. Given the growing body of evidence from psychometric, self-report studies for phenotypic overlap between ASCs and SSCs, and the ambiguities involved in interpreting their overlap in social phenotypes (mainly deficits), research that also uses genetic and neuropsychological data, to more directly assess causal factors, becomes especially useful. For example, Cheung and colleagues [89] compared regional grey matter volumes between ASCs and schizophrenia and found evidence for both shared and distinct neuroanatomical features between the two conditions, and Crespi and Crofts [90] describe evidence for both diametric and overlapping patterns in autism and schizophrenia with regard to copy-number variants.

Given current findings, as discussed here, the degree to which the overlapping social phenotypes of autism spectrum traits and schizotypy stem from diametric or overlapping causes, or both, remains an important question for future research. Generally, our results suggest that social-deficit and social-abnormality phenotypes, as assessed by scales such as the AQ and SPQ-BR, may have less usefulness in distinguishing between different psychological and psychiatric conditions than do positive phenotypes: differences *per se* in phenotypes, novel phenotypes, or increased expression of phenotypes, in contrast to negative phenotypes, which comprise general deficits, absences, unspecified abnormalities, or reductions [8]. Examples of such positive phenotypes for autism or schizotypy and schizophrenia would include magical thinking, hallucinations and delusions, jumping to conclusions, local-vs-global processing, insistence on sameness, enhanced visual-spatial skills, and attention to detail [8], [25]. In accordance with this approach, Russell-Smith and colleagues [91] found that higher levels of autistic features predicted enhanced performance in local versus global information processing as assessed by the Embedded Figures Test (EFT) [92], whereas positive schizotypal features predicted reduced performance. Our results also highlight the value in testing predictions from alternative models for the relationship between autism and schizotypy, rather than focussing on a single model and its predictions.
